# Biosensors for Detecting Lymphocytes and Immunoglobulins

**DOI:** 10.3390/bios10110155

**Published:** 2020-10-27

**Authors:** Pietro Salvo, Federico M. Vivaldi, Andrea Bonini, Denise Biagini, Francesca G. Bellagambi, Filippo M. Miliani, Fabio Di Francesco, Tommaso Lomonaco

**Affiliations:** 1 Institute of Clinical Physiology, National Council of Research, Via Moruzzi 1, 56124 Pisa, Italy; federicomaria.vivaldi@phd.unipi.it; 2 Department of Chemistry and Industrial Chemistry, University of Pisa, Via Moruzzi 13, 56124 Pisa, Italy; andrea.bonini@phd.unipi.it (A.B.); denisebiagini@virgilio.it (D.B.); f.miliani1@studenti.unipi.it (F.M.M.); fabio.difrancesco@unipi.it (F.D.F.); tommaso.lomonaco@unipi.it (T.L.); 3 Institut des Sciences Analytiques, UMR 5280, Université Lyon 1, 5, rue de la Doua, 69100 Villeurbanne, France; francesca.bellagambi@univ-lyon1.fr

**Keywords:** lymphocytes, immunoglobulins, B cells, T cells, natural killer cells, biosensors, immunosensors, aptasensors

## Abstract

Lymphocytes (B, T and natural killer cells) and immunoglobulins are essential for the adaptive immune response against external pathogens. Flow cytometry and enzyme-linked immunosorbent (ELISA) kits are the gold standards to detect immunoglobulins, B cells and T cells, whereas the impedance measurement is the most used technique for natural killer cells. For point-of-care, fast and low-cost devices, biosensors could be suitable for the reliable, stable and reproducible detection of immunoglobulins and lymphocytes. In the literature, such biosensors are commonly fabricated using antibodies, aptamers, proteins and nanomaterials, whereas electrochemical, optical and piezoelectric techniques are used for detection. This review describes how these measurement techniques and transducers can be used to fabricate biosensors for detecting lymphocytes and the total content of immunoglobulins. The various methods and configurations are reported, along with the advantages and current limitations.

## 1. Introduction

The adaptive immune response of the human body depends on the action of lymphocytes that respond to external antigens, such as viruses, bacteria and fungi. The number of lymphocytes is about 2 × 10^12^ and includes the B cells, the T cells and the natural killer (NK) cells [1].

B cells start developing in the bone marrow through three stages: pro-B, pre-B and immature B cells. Each B cell has a receptor that is specific for an antigen. Additionally known as hematogones, the immature B cells migrate to secondary lymphatic organs (e.g., spleen, lymph nodes and tonsils), where they bind to an antigen to become memory cells and effector cells [1,2,3]. The memory cells enhance and speed up the response of the immune system if the same foreign antigen enters the body again. The effector B cells produce antibodies to hinder the spread of antigens. B cells are about 3–21% of circulating lymphocytes. Antibodies are glycoproteins secreted by B cells when the immune system detects the presence of pathogens such as viruses. Antibodies are also known as immunoglobulins, which are divided in five main classes, namely IgG, IgA, IgM, IgE and IgD. IgG is the most abundant in human serum (about 70%). The basic structure of an IgG resembles that of a “Y”, where each arm consists of a heavy (about 50–70 kDa) and a light (about 25 kDa) polypeptide chain held together by disulfide bonds [4,5]. Each arm has a fragment antigen-binding (F_ab_) region that has a constant and a variable domain of amino acid sequences. The variable domain includes three regions called complementarity-determining regions (CDRs). The union of the heavy-chain CDRs with the light-chain CDRs form the paratope, which is capable of binding to the site (epitope) of an antigen [6]. A change in the length and sequence of the paratope allows the antibodies to be bound to a vast multitude of antigens [7].

T cells start developing in the thymus through three stages: pro-T, pre-T and immature T cells. Immature T cells migrate to secondary lymphatic organs unless they bind to self-antigens that induce apoptosis. As for B cells, after binding to antigens, T cells turn into memory and effector cells. T cells are about 51–88% of circulating lymphocytes and can be grouped into two categories, according to the presence of CD4^+^ or CD8^+^ antigens on their surfaces [1,2,3]. CD4^+^ T cells can be divided into helper (T_h_) and regulatory (T_reg_) T cells. T_h_ cells respond to an infection by activating macrophages, B cells and cytotoxic T cells (i.e., CD8^+^ T cells) and NK cells [8]. T_reg_ cells regulate the activity of T_h_ cells to avoid an uncontrolled and undesirable autoimmune response. CD8^+^ T cells not only kill pathogens but also play a role to regulate the autoimmune response [9]. A CD4/CD8 ratio < 1 is associated with inflammation, immune activation and immunosenescence, which lead to a high risk of morbidity and mortality [10]. NK cells are about 4–29% of circulating lymphocytes and do not need to be exposed to an antigen to start attacking pathogens, such as tumor- and virus-infected cells [11]. Table 1 shows the reference intervals in human serum for lymphocytes and immunoglobulins.

Relative lymphocytopenia occurs when the absolute total blood lymphocyte count is <1500/μL, whereas a count <1000/μL is associated with severe lymphocytopenia [12,13]. Lymphocytopenia is associated with several pathologies, such as immunodeficiency syndromes, Crohn’s disease, Sjögren’s disease, insulin-dependent diabetes mellitus, bone marrow hypoplasia, renal failure and cancer [12]. Lymphocytosis is the condition when the peripheral blood lymphocyte count exceeds about 4000–4500/μL, although this value is 8000/μL for young children [12,14]. Some conditions associated with lymphocytosis are lymphoid malignancies, hepatitis, varicella, tuberculosis, acute serum sickness, x-linked lymphoproliferative disease and thyrotoxicosis [12].

Therefore, the detection, quantification and characterization of lymphocytes and immunoglobulins play a fundamental role in clinical practice and scientific research. Flow cytometry is probably the most used technique in clinical laboratories for lymphocytes and immunoglobulin assessments, since it is relatively rapid, flexible and sensitive [15,16,17,18,19,20]. However, flow cytometry has some drawbacks and limitations, such as the lack of standardization in assays and instrument setup, laborious sample preparation and expensive equipment and reagents [21]. The development of easy-to-use, portable and affordable point-of-care (POC) devices would be a breakthrough in the analyses of lymphocytes and immunoglobulins. Techniques such as enzyme-linked immunosorbent assay (ELISA) are not presently suitable for POC devices or for resource-limited countries with a few specialized laboratories for clinical analyses [22,23]. Spectroscopy (e.g., Raman and near-infrared) and impedance measurements are currently under testing to fabricate reliable POC devices [16,17,24,25]. Biosensors could be a promising alternative to obtain compact, fast and low-cost devices for detecting lymphocytes and immunoglobulins. To this aim, the literature reports several examples of electrochemical, optical, field effect transistors (FETs) and piezoelectric biosensors. This review focuses on the most common biorecognition elements, such as antibodies, aptamers, nanomaterials and proteins, and detection techniques to discuss their advantages and drawbacks in biosensors for the detection and quantification of lymphocytes and total contents of human immunoglobulins.

## 2. B Cells and Immunoglobulins

### 2.1. Electrochemical Biosensors

In the literature, electrochemical biosensors are the most used to detect B cells and the immunoglobulins. In an amperometric biosensor, a constant voltage is applied to a working electrode, and the resulting steady-state current is measured. The current is proportional to the concentration of electroactive elements that oxidize or reduce at the surface of the working electrode. Since each electroactive element has a distinctive redox potential, amperometric biosensors have high selectivity [31]. Amperometric biosensors can be used in complex matrices and, thus, could be a valuable tool for a reliable clinical analysis.

An amperometric immunosensor with thiol-modified gold substrate was adopted for the detection of IgG in the human serum of patients affected by American trypanosomiasis [32]. The receptor was a horseradish peroxidase (HRP)-labeled goat anti-human IgG gamma chain (HRP-conjugated IgG). This sensor was comparable with ELISA but had false positives because of unspecific antigen binding. Another amperometric immunosensor consisted of screen-printed carbon electrodes for measuring IgE, which is known to trigger allergic disorders [33]. The enzyme p-aminophenyl phosphate (p-APP) was used as a mediator anchored to anti-IgE in a competitive assay. Although on a limited batch of blood samples, the results were comparable with ELISA and radioimmunoassay. Wang et al. improved the linear range for an amperometric immunosensor for IgG, fabricating a three-dimensional (3-mercaptopropyl)-trimethoxysilane sol–gel to absorb gold nanoparticles functionalized with anti-IgG. The enzyme HRP was used to block nonspecific bindings between antibodies and antigens. This immunosensor was tested in human serum and had a linear range of 1.12–162 ng/mL with a limit of detection (LOD) of 0.56 ng [34].

Differential pulse voltammetry (DPV) is a technique that applies potential pulses on a linear ramp potential and measures the current difference before and at the end of the applied pulse [35]. Due to diffusion, the current depends on molar concentrations in the range 10^−7^–10^−8^ mol/L. Papamichael et al. developed an aptasensor for IgE and obtained a LOD of 23 ± 4 ng/mL in a buffer solution [36]. In an aptasensor, the biorecognition element is an aptamer, i.e., a single-stranded deoxyribonucleic acid (DNA) or ribonucleic acid (RNA) oligonucleotide that can be immobilized at high density onto an electrode surface. An aptamer exhibits low immunogenicity and can recognize small molecules or whole cells with high specificity and affinity [37]. Since the selection of RNA aptamers entails reverse and in vitro transcription for each step, the large-scale production of RNA aptamers is more complex than DNA aptamers [38]. Wang et al. proposed an aptasensor with a zwitterionic peptide to reduce the nonspecific adsorption of IgE onto a gold electrode surface. In DPV experiments, this method led to a linear range of 0.1–10 pg/mL and a LOD of 42 fg/mL [39].

Li et al. fabricated an aptasensor where an aptamer was immobilized onto a gold surface and incubated in streptavidin-alkaline phosphatase (S-ALP) [40]. After adding IgE, the aptasensor was incubated in a 1-naphthyl phosphate (1-NP) solution. The enzymatic dephosphorylation of 1-NP catalyzed by S-ALP was reduced by IgE; thus, the decrease in the peak intensity could be associated with the concentration of the immunoglobulin. Salimi et al. [41] and Khezrian et al. [42] published two articles where a nanocomposite of multiwall carbon nanotubes, ionic liquid and chitosan (MWCNT/IL/Chit) was anchored onto a glassy carbon electrode to detect IgE by means of DPV. MWCNTs have a high surface-to-volume ratio, whereas IL prevents the nanotube aggregation. Chitosan is a polysaccharide with reactive amino and hydroxyl functional groups that is often used to improve the immobilization of molecules [41]. In [41], the MWCNT/IL/Chit nanocomposite was used in a sandwich assay with two aptamers (5′-amine-terminated and an aptamer modified with biotin and HRP), whereas, in [42], methylene blue (MB) competed with IgE to bind to an aptamer linked to the nanocomposite. The sandwich approach had the best performances with a linear range from 50 pM to 2 nM and a LOD of 6 pM. 

**Figure 1:**
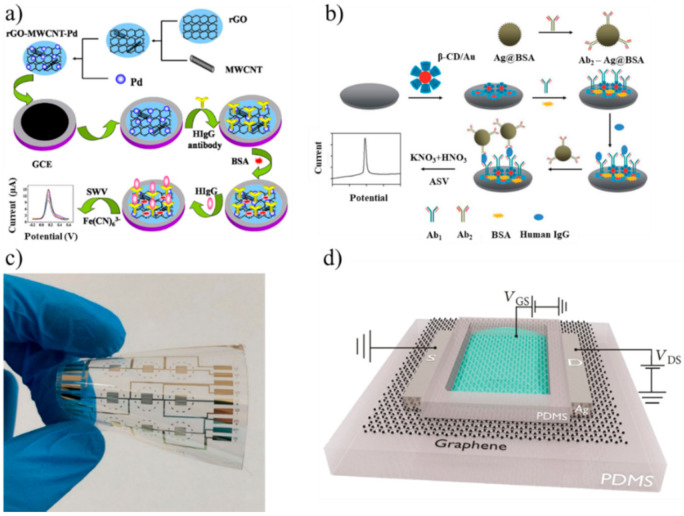
(**a**) Fabrication of an immunosensor for immunoglobulin G (IgG) using a reduced graphene oxide-multiwall carbon nanotubes-palladium NP (rGO–MWCNT–Pd) nanocomposite (adapted with permission from [47], Copyright Elsevier 2015). (**b**) Fabrication of an immunosensor immobilized onto bovine serum albumin (BSA)-stabilized silver microspheres (Ag@BSA) (adapted from [50] with permission from The Royal Society of Chemistry). (**c**) Gold interdigitated electrodes on flexible polyethylene naphthalate (PEN) for detecting IgG (adapted from [61] under Creative Commons CC BY 4.0 license). (**d**) Simplified view of a graphene-based field effect transistor aptasensor (adapted from [65] under Creative Commons CC BY 4.0 license). SWV: square wave voltammetry and HIgG: human IgG.

Other authors obtained similar results using aptasensors that conjugated aptamers with nanoparticles (NPs). NPs can facilitate the electron transfer from the redox center of a protein to the electrode surface [43]. The simplest NP aptasensor was a sandwich assay with an aptamer-gold NP conjugate (AuNPs) where IgE competed with MB. The DPV measurements showed a LOD of 0.52 ng/mL [44]. Lee et al. used square wave voltammetry (SWV) to detect IgE using an aptamer immobilized onto AuNPs and amplifying the signal by a guanine-rich complementary DNA (cDNA G1) [45]. The cDNA G1 bound to the aptamer; however, when the aptamer captured human IgE, the binding was inhibited, and the level of IgE could be measured (LOD 0.16 pM). The SWV combines a large amplitude square wave modulation with a staircase waveform. The signal is obtained as the difference between the currents measured at the end of the direct and reverse pulses [46]. Liu et al. applied SWV to measure the concentration of IgG in human serum [47]. The immunosensor consisted of a glassy carbon electrode (GCE) coated with a reduced graphene oxide–MWCNT–(palladium NPs) nanocomposite (rGO–MWCNT–Pd) that was used to immobilize anti-IgG (Figure 1a). The MWCNTs avoided the aggregation of rGO, which is a good electrical conductor, and improved the surface area. This immunosensor had a linear range of 0.01–25 ng/mL and LOD of 3.3 pg/mL.

Song et al. used a sandwich strategy for measuring IgE using a thiol-capped aptamer as a capture probe, whereas the detection probe consisted of anti-human IgE antibodies linked to a nanocomposite of silver NPs and graphene functionalized with streptavidin (LOD 3.6 ng/mL) [48]. The measurement was performed using square wave anodic stripping voltammetry (SWASV), a technique where the analyte of interest is electroplated on the working electrode during a deposition step. The current is measured during the stripping step (a square wave) when the analyte is removed from the electrode [49]. Zhang et al. chose ASV to detect IgG in human serum [50]. The electrode surface was coated with β-cyclodextrin-functionalized AuNPs (β-CD/Au). Bovine serum albumin (BSA)-stabilized silver microspheres (Ag@BSA) were chosen, since they can be electrochemically oxidized at a low potential to yield a well-shaped stripping peak. A sandwich assay with anti-IgG anchored onto β-CD/Au and Ag@BSA completed the immunosensor (Figure 1b). The linear range was 0.001–10 pg/mL with a LOD of 0.5 fg/mL. Jiang et al. designed and produced a pseudoknot aptamer bound to AuNPs to achieve a LOD of 60 pM using SWV in human serum [51]. The results with NPs and voltammetry suggest that nanocomposites have the potential to be used in clinical practice, although more tests on repeatability and stability are needed. 

Cyclic voltammetry (CV) is a technique that measures the current when the potential is scanned during an experiment. The Nernst equation defines how the concentration of the species in solution near the working electrode changes over time [52]. Oh et al. applied CV to an immunosensing platform for detecting IgE in multiple samples simultaneously [53]. A gold electrode surface was treated with a mixed self-assembled monolayer (SAM) containing 11-mercapto-1-undecanol (MUO), 12-mercaptododecanoic acid (MDA) and 11-(mercaptoundecyl)ferrocene (MUF). The hydroxyl group of MUO prevented nonspecific binding; the carboxylic group of MDA improved the immobilization of the antibodies, whereas the ferrocene moiety of MUF improved the electron transfer of the reaction. Exploiting the catalytic activity of the enzyme ALP conjugated to the secondary antibody in a sandwich assay, this immunosensor achieved a LOD of 1.45 ng/mL. However, the authors pointed out that reproducibility and the signal-to-noise ratio (SNR) should be improved to use this immunosensor as an analytical tool.

#### 2.1.1. Impedimetric Biosensors

Impedimetric measurements usually consist of monitoring the electrical changes that occur in a biological system. A simple approach was adopted by Hianik et al., who measured the conductivity when IgE bound to swine anti-human IgE immobilized through cysteamine to a gold-coated silicon substrate [54]. This immunosensor had a range of about 1–180 nM (LOD of 1 nM), but it was not clear whether this result depended on an interaction antigen-antibody. In fact, the addition of BSA as a blocking agent changed the sign of the response and led to a nonlinear behavior. Other authors investigated the functionalization of nanowires, which have a high surface-to-volume ratio and high sensitivity because of negligible lateral current shunting [55]. Although tested in phosphate-buffered solution (PBS), polymer nanowires showed interesting results for the detection of IgG. The polymers were polyaniline (PANI) or a mixture of poly-pyrrole (Ppy) and poly(ethylene oxide). Anti-IgG were immobilized onto the nanowires, and a conductivity change was observed after the interaction with the antigen [56,57]. PANI and Ppy are *p*-type conductive polymers, thus conductivity increase when IgG accumulates on the nanowire surface, since IgG (pI 7.2) has a weak negative charge in PBS (pH 7.4).

Labib et al. developed a capacitive biosensor where concanavalin A (Con A) was covalently attached to SAMs of carboxylic acid terminated alkyl-thiols onto a gold working electrode in a three-electrode setup [58]. Con A is a tetrameric metalloprotein that binds IgG. The capacitance at the working electrode surface was the sum of three contributions: the capacitance of thiol groups, the capacitance of the IgG layer and the double-layer capacitance. This biosensor showed a LOD of 1 µg/mL with good reproducibility and the possibility to be regenerated for further use. 

A deep electrical analysis of an electroactive biological system can be performed by means of electrochemical impedance spectroscopy (EIS). EIS is a nondestructive technique that measures the electrical impedance of a biological system when a small voltage is applied. The electrochemical impedance (Z) depends on several factors that can be included in an equivalent electrical circuit. The simplest equivalent circuit is the Randles cell, which consists of the solution resistance (R_s_), the double-layer capacitance at the electrode surface (C_dl_), the charge transfer resistance (R_ct_) and the impedance due to diffusion (Warburg impedance) [59]. The real and imaginary parts of Z are usually plotted in a Nyquist plot. In an electrochemical cell, R_ct_ is typically measured, since it changes because of biological reactions such as the binding antibody-antigen at the working electrode surface. Qi et al. performed EIS experiments to detect IgG in PBS using protein A as the recognition element [60]. Protein A is found on the cell walls of *Staphylococcus aureus* bacterial strains, and it can specifically bind to human IgG. This biosensor had a linear range of 10–1000 ng/mL with a LOD of 5 ng/mL and proved selective for IgG when tested with interferents such as BSA and IgA. A smaller linear range (0.01–10 ng/mL) but better LOD (0.01 ng/mL) for IgG was obtained in PBS during EIS experiments with an immunosensor with interdigitated gold electrodes (IDEs) on a flexible polyethylene naphthalate (PEN) substrate (Figure 1c) [61]. Compared with the classical electrode design, IDEs offer some advantages in biosensing, such as improved sensitivity, SNR and the aspect ratio. Ohno et al. adopted EIS and IDEs to measure IgA (a biomarker for pathologies such as depressive disorders and nephropathy) in an immunosensor that had a linear range of 0.01–100 ng/mL and LOD of 0.01 ng/mL [62].

#### 2.1.2. Field Effect Transistors

A field effect transistor (FET) is a device that controls the current flowing in a semiconductor between two terminals (source and drain) through the voltage applied to a third terminal (gate). For biosensing, the gate surface is usually modified to react with the analyte of interest. The chemical reaction modifies the minimum gate voltage (threshold voltage, V_T_) that allows the current to flow into the FET. Therefore, a change in V_T_ (current) can be associated with the concentration of the targeted analyte after calibration. FETs are suitable for mass production at low costs per piece, are potentially fast and ultra-sensitive, can be miniaturized (the gate length, i.e., the channel length where the current flows between source and drain; can be in the order of nm) and offer a compact multi-sensor platform.

In 2008, Cid et al. used chemical vapor deposition (CVD) to grow a single-wall CNT (SWCNT) network between a source and drain. SWCNTs were functionalized with anti-IgG to detect IgG. This FET worked in the mg/L range and showed that this label-free technology could be used for measuring immunoglobulins [63]. In the same year, Maehashi et al. used a similar approach, using aptamers for the detection of IgE and achieved a linear range of 250 pM–160 nM [64]. Recently, Lan et al. kept the aptasensor approach but substituted CNTs with graphene to improve the FET electrical properties (Figure 1d) [65]. After increasing the volume of 1-μM IgE from 0 to 30 μL (5-μL steps), the current increased by about 10^−7^ mA after each addition.

Hayashi et al. measured the concentration of IgA in sweat samples using a plant lectin, jacalin, as the receptor [66]. The SiO_2_ gate surface was functionalized with 3-Aminopropyl)triethoxysilane (APTES) and glutaraldehyde to anchor jacalin. Ethanolamine blocked the nonspecific binding of IgA. Sweat samples had to be filtered before analysis, and the authors explained that further treatments are needed to decrease contaminants.

Table 2 summarizes the main characteristics of the electrochemical biosensors to detect immunoglobulins.

### 2.2. Optical Biosensors

Surface plasmon resonance (SPR) is the oscillation of an electromagnetic wave at the interface of two media with dielectric constants of opposite signs [67]. SPR is a popular technique to measure the adsorption of molecules onto the metallic surface, since the plasma wave is very sensitive to the changes of the optical properties of the dielectric in contact with the metallic film.

In 2009, Kim et al. functionalized a gold film with carboxylic- and hydroxyl-terminated SAM containing ethylene glycols. After immobilizing streptavidin onto the SAM layer, an aptamer was anchored to streptavidin to detect IgE [68]. This biosensor had a LOD of about 2 ng/mL and discriminated between IgE and other proteins such as IgG and fibrinogen. Sriwichai et al. fabricated an immunosensor where poly(3-aminobenzoic acid) (PABA) was used to immobilize anti-IgG [69]. This immunosensor was tested with SPR in a buffer and achieved a LOD of 1 µg/mL; however, repeatability tests are needed to assess its performance. Instead of antibodies and aptamers, Ertürk et al. used molecularly imprinted F_ab_ fragments to detect IgG. This biosensor had a linear range of 0.02–1 mg/mL and was capable of detecting a concentration of about 0.6-µg/mL IgG in human plasma [70]. Other authors effectively used SPR to measure the concentration of IgG. The receptor changed from a hen egg lysozyme [71] to recombinant staphylococcal protein A [72,73]. Protein A was also used in a label-free photonic crystal biosensor for detecting IgG [74]; however, the promising results of protein A need to be validated in real samples.

Liu et al. developed an electrochemiluminescence (ECL) aptasensor to detect IgE [75]. CdSe/ZnS quantum dots (QDs) were functionalized with MoS_2_, a metal dichalcogenide, and the enzyme-induced catalyzed biocatalytic precipitation (BCP) of HRP was used for signal quenching. An aptamer was immobilized onto a glassy carbon electrode coated with MoS_2_ QDs. A competitive assay was performed using an aptamer bound to the composite AuNPs-HRP. This aptasensor was tested in human serum and had an error less than 10% compared with ELISA. Another group measured the photoluminescence (PL) of a heterometallic cluster, (Ag_6_Au_6_(ethisterone)12)-estrogen receptor α (Ag_6_Au_6_Eth-ERα), using graphene oxide (GO) as quencher [76]. The PL was restored with IgG, which could be thus measured in a buffer solution.

**Figure 2:**
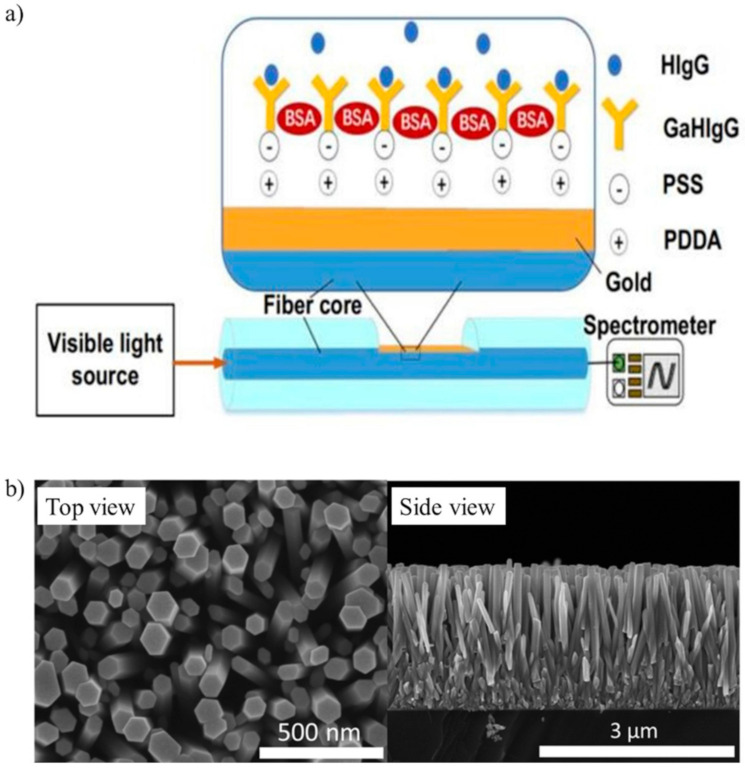
(**a**) Optical D-type fiber immunosensor functionalized with poly dimethyl diallyl ammonium chloride (PDDA) and poly(sodium-p-styrenesulfonate) (PSS) to immobilize goat anti-human IgG (GaHIgG) for detecting human IgG (HIgG) (adapted with permission from [79], Copyright Elsevier 2020). (**b**) Top and side SEM images of SiO_2_/ZnO nanowires (adapted from [88] with permission from The Royal Society of Chemistry).

In the literature, optical fibers have often been used as biosensors, because they are low-cost, have excellent light delivery, long interaction lengths and can capture the emitted light from the target analytes [77]. Typically, optical fibers have a functionalized extremity to detect the analyte of interest. For the detection of IgG, Wang et al. functionalized an optical fiber with staphylococcal protein A and goat anti-human IgG by covalent binding [78]. A Mach-Zender interferometer allowed IgG to be detected with a LOD of 47 ng/mL in PBS. Another biosensor proposed a D-type fiber, which was functionalized with poly dimethyl diallyl ammonium chloride (PDDA) and poly(sodium-p-styrenesulfonate) (PSS) before immobilizing anti-human IgG (Figure 2a) [79]. This optical fiber was tested in a buffer solution and had good specificity against gelatin, horse IgG and swine IgG.

Table 3 shows the main characteristics of the optical biosensors to detect immunoglobulins. Most of the linear ranges and LODs are within the clinical range of interest, but it is worth noting that most of the biosensors were tested in a buffer. Although a few biosensors were tested for interferents, tests in complex matrices such as plasma or serum are mandatory for practical use.

### 2.3. Piezoelectric Biosensors

Piezoelectricity is the ability of a material to generate an internal electric field when a mechanical stress or strain (direct piezoelectric effect) is applied and vice versa (inverse piezoelectric effect). Piezoelectric materials are usually crystals, ceramics and polymer films, such as aluminum phosphate, aluminum nitride, zinc oxide, quartz (SiO_2_), tartrate tetrahydrate (Rochelle salt), polyvinylidene fluoride and polylactic acids [80]. In biosensing, an alternating voltage is applied to a piezoelectric material between two electrodes. When the analyte of interest interacts with the surface of an electrode (e.g., an antigen that binds to an antibody immobilized onto the electrode surface), the oscillation frequency of the piezo material changes according to the mass bound to the electrode [81] or to the medium viscosity [82].

In 1994, a piezoelectric immunosensor with a 10-MHz AT-cut crystal resonator (quartz crystal microbalance, QCM) was proposed for counting B cells. Anti-B cells were immobilized onto the electrode surface using polyethyleneimine, which proved more sensitive than protein A and APTES in whole human blood [83]. Chu et al. [84] and Su et al. [85] adopted the QCM-immunosensor approach to detect IgM and IgE in serum samples, respectively, whereas Pohanka measured the concentration of IgG in human plasma [86]. A QCM was also used to measure the IgA concentration in human saliva, with the results comparable with ELISA. However, repeatability, stability and specificity tests were not reported [87].

Zhao et al. proposed nanowires to detect IgG in a buffer solution [88]. Briefly, piezoelectric ZnO nanowires were vertically grown onto a Ti plate (first electrode) and then coated with a SiO_2_ layer. The surfaces of SiO_2_/ZnO nanowires were modified with AuNPs and anti-IgG. An Al foil on top of the nanowires was the second electrode. The adsorption of antigens modified the surface free-carrier density on the surface of the nanowires. Therefore, this change in the free-carrier density could be used to monitor the concentration of antigens. Figure 2b shows the scanning electron microscope (SEM) images of the nanowires.

Other authors used piezoelectricity to fabricate flexural plate wave (FPW) biosensors for immunoglobulins. Typically, a FPW device consists of a ZnO film on interdigitated electrodes. When the film undergoes a mechanical deformation, an acoustic wave propagates within the device. However, the acoustic wave is sensitive to any changes on the film surface. Therefore, amplitude, frequency and phase of the wave can be measured to detect the binding of immunoglobulins [89]. In the literature, the same group developed three FPW devices to measure the concentration of IgE [90,91,92], but only the most recent was tested in human serum, although no data were reported on reproducibility and stability.

Table 4 shows the main characteristics of the piezoelectric biosensors to count B cells and detect immunoglobulins. These biosensors were mostly tested in real samples, and QCM could be a reliable alternative to ELISA if stability and reproducibility tests will confirm these preliminary results.

## 3. T Cells

### 3.1. Electrochemical Biosensors

**Figure 3:**
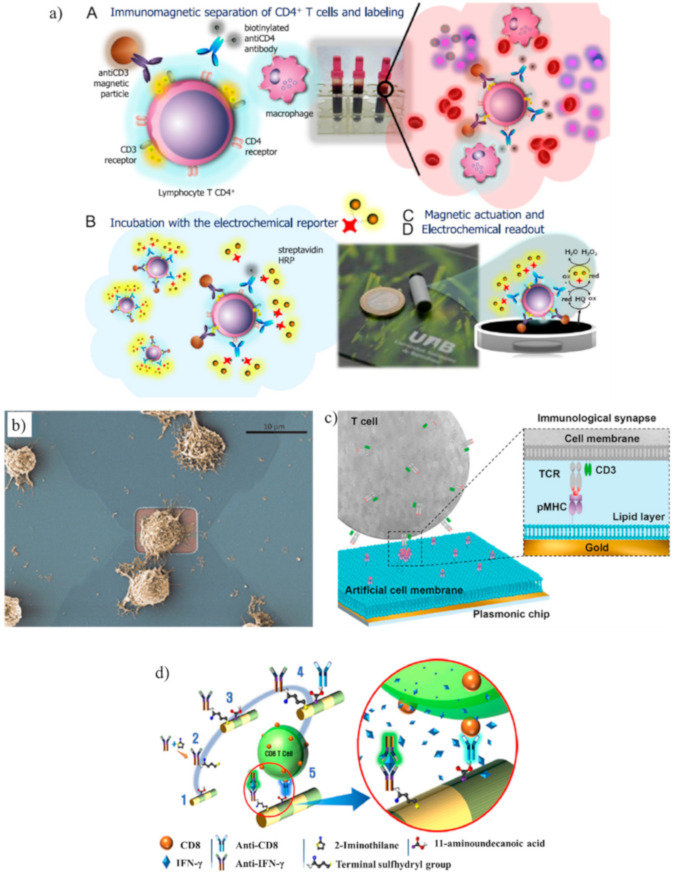
(**a**) Schematic representation of a magneto-actuated biosensor where commercial magnetic particles were modified with an anti-CD3 antibody to isolate CD4^+^ cells from monocytes and macrophages. Biotinylated anti-CD4 antibodies labeled with streptavidin-horseradish peroxidase (HRP) were used to label the isolated CD4^+^ cells. The amperometric measurement was mediated by H_2_O_2_ and hydroquinone (adapted with permission from [93], Copyright Elsevier 2015). (**b**) Example of a cytotoxic T cell adhered on top of a transistor gate with a dimension of 12 × 5 mm^2^ (adapted with permission from [99], Copyright Elsevier 2015). (**c**) Schematic representation of a label-free Surface plasmon resonance (SPR) biosensor to analyze tumor-specific CD8^+^ cells. The gold layer of the SPR sensor was coated with planar lipid bilayers to immobilize major histocompatibility complex with antigen-derived peptides (p/MHC) that bound with CD8^+^ cells (TCR, T cell receptor) (adapted with permission from [102], Copyright 2018 American Chemical Society). (**d**) Schematic representation of barcode nanowires of alternated Fe and Au multilayers to capture CD8^+^ cells and their secreted interferon-γ (IFN-γ). The iron segment was treated with 11-aminoundecanoic acid for binding anti-CD8, whereas thiolated anti-IFN-γ antibodies were conjugated to the Au segment (adapted with permission from [105], Copyright 2019 American Chemical Society).

CD4 and CD3 receptors are expressed simultaneously only by CD4^+^ lymphocytes, whereas monocytes and macrophages only express the CD4 receptor. Carinelli et al. exploited commercial magnetic particles modified with an antiCD3 antibody to isolate CD4^+^ cells from monocytes and macrophages. Biotinylated antiCD4 antibodies labeled with streptavidin-HRP were used to label the isolated CD4^+^ cells. H_2_O_2_ and hydroquinone were used to perform an amperometric detection (Figure 3a) [93]. Kim et al. found that two-step oxygen plasma could improve the performance of a SWCNT immunosensor for CD4^+^ lymphocytes. The first plasma treatment cleaned the nanotubes from fabrication leftover, whereas the second treatment generated carboxyl groups that improved the immobilization of antibodies [94].

Two groups proposed the impedance measurement for CD4^+^ cells [95,96]. In deionized water, Wang et al. used a label-free microfluidic chip that could detect CD4^+^ cells with a LOD of 10 cells/μL [96]. The impedance-based label-free cellular assay was also used in the commercial xCELLigence system, which could monitor T cell activation in real time [97].

A gold nanoparticles-doped polyaniline nanofiber (Au/PANI-NF) composite was synthesized to monitor the activation of T cells at early, middle and late stages [98]. Anti-CD were immobilized onto the composite to monitor the expression of CD69, CD25 and CD71, which are T-cell surface activation markers. EIS measurements showed a LOD of 10^4^ cells/mL. This sensor had a higher LOD than other electrochemical biosensors for monitoring T cells; however, it was the first work using EIS, and improvements are possible.

A notable work to study CD8^+^ cell adhesion and migration was performed by Law et al. [99]. Although the purpose was not to count or detect T cells, the authors fabricated FETs that achieved single-cell measurements (Figure 3b). The activation of CD8^+^ cells is essential for the activation of cytotoxic T lymphocytes. The results showed that CD8^+^ cells had a stronger adhesion to anti-CD3 antibodies than anti-LFA-1 antibodies (LFA-1 is an integrin that has an important role in the activation of cytotoxic T cells).

### 3.2. Optical Biosensor

In the peripheral blood of uremic patients, levels of CD4^+^ are reduced. Wang et al. compared the SPR responses of CD4^+^ cells in healthy and hemodialyzed volunteers to prove that SPR could successfully discriminate the two groups [100]. Another method to detect CD4^+^ cells consisted of tagging with fluorescent labels major histocompatibility complex (MHC) proteins loaded with antigen-derived peptides (p/MHC) [101]. The combination of grating-coupled surface plasmon resonance imaging (GCSPRI) and grating-coupled surface plasmon coupled emission (SPCE) was investigated for the detection of CD4^+^ cells bound to p/MHC. The evanescent wave of the GCSPRI system only extends about 200 nm above from the surface where CD4^+^ cells bind to p/MHC. Therefore, only the cells that are on the surface contribute to the output signal, which SPCE amplified to readable levels. However, MHC proteins are polymorphic; thus, MHC must be determined for each patient to select the most suitable fluorescent label.

The detection of the interaction between T cells and p/MHC was improved using a label-free SPR biosensor to analyze tumor-specific CD8^+^ cells expressing engineered receptors for the melanoma antigen NY-ESO-I [102]. The gold layer of the SPR sensor was coated with an artificial cell membrane formed by planar lipid bilayers that served for the immobilization of p/MHC (Figure 3c). SPR measured the concentration of CD8^+^ cells in the range 10^2^–10^5^ cells/mL. The same device detected the concentration of T cells (SupT1 cells, i.e., T lymphoblasts) using antiCD3 antibodies with a LOD of 500 cells/mL.

Thorslund et al. fabricated polydimethylsiloxane (PDMS) microchannels to count CD4^+^ cells in blood using a combination of fluorescein isothiocyanate (FITC)-conjugated mouse anti-human CD3 antibody, anti-human CD4 antibody and a DNA-binding fluorochrome (HOECHST 33342) [103]. The main achievement of this work was the coating of the hydrophobic PDMS microchannel with a hydrophilic heparin layer. This coating allowed blood to be drawn by capillarity and avoided red blood cell adhesion to PDMS. Furthermore, this coating allowed the microchannel to be coated with avidin, which was used to immobilize the complex biotin-CD4 antibody.

Xiao et al. counted CD4^+^ cells using fluorescent immuno-chromatographic strips (ICSs) that measured the free CD4^+^ antibodies not bound by CD4^+^ cells [104]. In whole-blood samples, ICSs exhibited a linear range of 100–800 cells/μL and LOD of 44 cells/μL. The cost per ICS was estimated at 0.5 USD, which is the less expensive biosensor among those reported in the literature for the same application.

Excellent performances in the capturing of CD8^+^ cells and their secreted interferon-γ (IFN-γ) were achieved using multilayered nanowires, which are called barcode nanowires (BNWs) [105]. BNWs consisted of alternately stacked iron and gold layers. The iron segment was treated with 11-aminoundecanoic acid to activate the initial functional group for binding anti-CD8, whereas thiolated anti-IFN-γ antibodies were conjugated to the Au segment (Figure 3d). The capturing of CD8^+^ cells and IFN-γ was confirmed by flow cytometry.

A device that could detect both CD4^+^ and CD8^+^ cells was proposed by Gohring and Fan, who developed an optofluidic ring resonator (OFRR) [106]. An OFRR is a tapered fiber where light is confined into the wall in circular resonance. At resonance with the incident light, the intensity shows an abrupt decrease. The evanescent field extends into the microfluidic channel and interacts with the antigens bound to antibodies for about 100 nm from the inner interface. This OFRR detected CD4^+^ and CD8^+^ cells in the ranges 160–300 cells/µL and 250–1000 cells/µL, respectively.

Table 5 summarizes the main characteristics of the biosensors for detecting T cells.

## 4. NK Cells

In the literature, the detection of NK cells was mainly performed by impedance analysis. In 2006, Glamann and Hansen added NK cells to multi-well plates seeded with MCF-7 cells (i.e., epithelial adenocarcinoma cells derived from a patient diagnosed with breast cancer) adhered to the surface [107]. The addition of NK cells changed the impedance measured at the bottom of each well and could be associated with the cells number, which is expressed as a dimensionless cell index (CI). The same method was used in [108] using fibroblasts (NIH 3T3) and other cancer cell lines such as H460 and HepG2. The commercial xCELLigence system adopts this approach using E-plates with gold electrodes at the bottom of a well to detect the cell morphology, adherence and cell index in real time [109,110,111].

## 5. Conclusions

The development of a biosensor for monitoring immunoglobulins or lymphocytes is challenging. Most of the works in the literature do not perform any experiment in real samples, such as serums or whole blood. Impedimetric and FET biosensors for detecting immunoglobulin are not yet mature enough to be tested in clinical practice, although there were some works that achieved a suitable clinical range of detection. The continuous research in 2D materials could boost the performance of FETs. However, although FET technology is well-consolidated in the manufacturing of electronic devices, FET devices still lack stability and reproducibility in biosensing applications. These limitations could be overcome, improving the electrical response with 2D materials and updating the protocols to avoid nonspecific binding. Optical biosensors for immunoglobulins were mostly based on SPR detection; however, almost all of them only worked in buffer solutions. Piezoelectric biosensors could compete with voltammetry-based techniques and propose QCMs as a valid alternative to existing laboratory techniques. In complex matrices, voltammetry-based techniques provided the best response, but it was not clear if antibodies or aptamers were the most suitable receptors for an immunoglobulin sensor. Aptamers have several advantages compared with antibodies, such as a longer shelf life, lower production cost, high batch-to-batch reproducibility and reversible heat-denaturation. However, aptamers have a short half-life in vivo or when exposed to nucleases unless they are chemically modified.

Similar conclusions can be drawn for T cell biosensors where, however, the state of the art is fuzzier. In fact, no technology stands out against the others. In some cases, the biosensors seem too complex to be integrated into POC devices. NK cell biosensors probably suffer from the competition with the xCELLigence system. The impedance measured when NK cells interact with other cells is an established and relatively simple technology that offers reliable results.

However, scientific research should be encouraged to develop more compact and low-cost devices. The future of these biosensors will depend on the results that researchers will be able to provide in tests with real samples. In particular, if the stability against nuclease is improved, aptamers may be the best receptor for future biosensors.

## Figures and Tables

**Table 1:** Reference intervals for lymphocytes and immunoglobulins in human serum.

Lymphocytes/Immunoglobulins	Normal Range	Reference
Total lymphocyte count	1054–3139 cells/µL	[26]
B cells	87–536 cells/µL	[26]
T Cells	17–2272 cells/µL	[26]
NK cells	93–840/mL cells/µL	[26]
IgG	8.19–17.25 mg/mL	[26]
IgA	0.70–3.86 mg/mL	[26]
IgM	0.55–3.07 mg/mL	[26]
IgE	2.4–240 ng/mL	[27,28]
IgD	5–240 mg/L ^1^	[29]
CD4	376–1292 cells/µL	[26]
CD8	216–1100 cells/µL	[26]
CD4/CD8 ratio	1–4	[10,30]

^1^ The concentration of IgD in the serum is highly variable among individuals. Ig: immunoglobulin and NK: natural killer.

**Table 2:** Characteristics of the electrochemical biosensors for detecting immunoglobulins (N.A. not available).

Target	Method of Detection	Receptor ^1^	Linear Range	Limit of Detection	Medium	Reference
IgG	Amperometry	Antibody	N.A.	N.A.	Serum	[32]
IgE	Amperometry	Antibody	100–1500 ng/mL	0.09 ng/mL	Plasma	[33]
IgG	Amperometry	Antibody	1.12–162 ng/mL	0.56 ng/mL	Serum	[34]
IgE	DPV	Aptamer	N.A.	23 ± 4 ng/mL	Buffer	[36]
IgE	DPV	Aptamer	0.1–10 pg/mL	42 fg/mL	Serum	[39]
IgE	DPV	Aptamer	~4 × 10^−6^–4 × 10^−1^ μg/mL	~4 × 10^−6^ μg/mL	Serum	[40]
IgE	DPV	Aptamer	50 pM–2 nM	6 pM	Serum	[41]
IgE	DPV	Aptamer	0.5–30 nM	37 pM	Serum	[42]
IgE	DPV	Aptamer	1–10,000 ng/mL	0.52 ng/mL	Buffer	[44]
IgE	SWV	Aptamer	1–100,000 pM	0.16 pM	Buffer	[45]
IgG	SWV	Antibody	0.01–25 ng/mL	3.3 pg/mL	Serum	[47]
IgE	SWASV	Aptamer	10–1000 ng/mL	3.6 ng/mL	Buffer	[48]
IgG	ASV	Antibody	0.001–10 pg/mL	0.5 fg/mL	Serum	[50]
IgE	SWV	Aptamer	0.1–100 nM	60 pM	Serum	[51]
IgE	Conductivity	Antibody	1–180 nM	1 nM	Buffer	[54]
IgG	Conductivity	Antibody	3 ng/mL–3 µg/mL	3 ng/mL	Buffer	[56]
IgG	Conductivity	Antibody	N.A.	N.A.	Buffer	[57]
IgG	Capacitive biosensor	Con A	5.0–100 mg/mL	1 µg/mL	Buffer	[58]
IgG	EIS	protein A	10 ng/mL–1 μg/mL	5 ng/mL	Buffer	[60]
IgG	EIS	Antibody	0.01–10 ng/mL	0.01 ng/mL	Buffer	[61]
IgA	EIS	Antibody	0.01–100 ng/mL.	0.01 ng/mL	Buffer	[62]
IgG	FET	Antibody	N.A.	1.25 mg/L	Buffer	[63]
IgE	FET	Aptamer	250 pM–160 nM	N.A.	Buffer	[64]
IgE	FET	Aptamer	N.A.	N.A.	Buffer	[65]
IgA	FET	Jacalin	1–100 μg/mL	1 μg/mL	Sweat	[66]

^1^ All the aptamers were DNA aptamers. DPV: differential pulse voltammetry, SWV: square wave voltammetry, SWASV: square wave anodic stripping voltammetry, ASV: anodic stripping voltammetry, EIS: electrochemical impedance spectroscopy, FET: field effect transistor and Con A: concanavalin A.

**Table 3:** Characteristics of the optical biosensors for detecting immunoglobulins (N.A., not available).

Target	Method of Detection	Receptor ^1^	Linear Range	Limit of Detection	Medium	Reference
IgE	SPR	Aptamer	1–1000 ng/mL	2 ng/mL	Buffer	[68]
IgG	SPR	Antibody	1–10 µg/mL	1 µg/mL	Buffer	[69]
IgG	SPR	F_ab_ fragment	0.02–1 mg/mL	N.A.	Plasma	[70]
IgG	SPR	Hen egg lysozyme	N.A.	N.A.	Buffer	[71]
IgG	SPR	Protein A	2–10 μg/mL	N.A.	Buffer	[72]
IgG	SPR	Protein A	30–100 μg/mL	0.5 μg/mL	Buffer	[73]
IgG	Photonic crystal	Protein A	0.5–10 mg/mL	N.A.	Buffer	[74]
IgE	ECL	Aptamer	0.5 pM–0.5 nM	0.18 pM	Serum	[75]
IgG	PL	Ag_6_Au_6_Eth-ERα	0.0078–10 ng/mL	0.65 pg/mL	Buffer	[76]
IgG	Interferometry	Protein A and anti-IgG	0.5–5 μg/mL	47 ng/mL	Buffer	[78]
IgG	SPR (optical fiber)	Antibody	0.02–0.08 mg/mL	0.2 μg/ml	Buffer	[79]

^1^ All the aptamers were DNA-aptamers. SPR: surface plasmon resonance, ECL: electrochemiluminescence, PL: photoluminescence, F_ab_: fragment antigen-binding and Ag_6_Au_6_Eth-Erα: (Ag_6_Au_6_(ethisterone)12)-estrogen receptor α.

**Table 4:** Characteristics of the piezoelectric biosensors for detecting B cells and immunoglobulins (N.A., not available). QCM: quartz crystal microbalance and FPW: flexural plate wave.

Target	Method of Detection	Receptor	Linear Range	Limit of Detection	Medium	Reference
B cells	Piezoelectricity, QCM	Antibody	5 × 10^3^–5.6 × 10^5^ cells	N.A.	Whole blood	[83]
IgM	Piezoelectricity, QCM	Antibody	5–93 µg/mL	N.A.	Serum	[84]
IgE	Piezoelectricity, QCM	Antibody	5–300 IU/mL	N.A.	Serum	[85]
IgG	Piezoelectricity, QCM	Antibody	0.0390–20 mg/mL	9.7 μg/mL	Plasma	[86]
IgA	Piezoelectricity, QCM	Antibody	1–3 µg/mL	N.A.	Saliva	[87]
IgG	Piezoelectricity, nanowires	Antibody	10^−8^–10^−3^ g/mL	5.7 ng/mL	Buffer	[88]
IgE	FPW	Antibody	~0.09–2.8 µg/mL	N.A.	Buffer	[90]
IgE	FPW	Antibody	N.A.	N.A.	Buffer	[91]
IgE	FPW	Antibody	N.A.	N.A.	Serum	[92]

**Table 5:** Characteristics of the biosensors for detecting T cells (N.A., not available).

Target	Method of Detection	Receptor	Linear Range	Limit of Detection	Medium	Reference
CD4^+^	Amperometry	Antibody	89–912 cells/μL (logistic)	44 cells/μL	Blood	[93]
CD4^+^	SWV	Antibody	10^2^–10^6^ cells/mL	10^2^ cells/mL	Buffer	[94]
CD4^+^	Impedance	Antibody	N.A.	N.A.	Blood	[95]
CD4^+^	Impedance	– ^1^	10–3000 cells/μL	10 cells/μL	DI water	[96]
T cells	EIS	Antibody	N.A.	10^4^ cells/mL	Buffer	[98]
CD8^+^	SPR	p/MHC	10^2^–10^5^ cells/mL	N.A.	Buffer	[102]
T cells	SPR	Antibody	10^2^–10^5^ cells/mL	500 cells/mL	Buffer	[102]
CD4^+^	Fluorescence	Antibody	100–800 cells/μL	44 cells/μL	Blood	[104]
CD4^+^	OFRR	Antibody	160–300 cells/µL	N.A.	Buffer	[106]
CD8^+^	OFRR	Antibody	250–1000 cells/µL	N.A.	Buffer	[106]

^1^ No receptor was used. OFRR: optofluidic ring resonator and p/MHC: major histocompatibility complex with antigen-derived peptides.
